# Safety and efficacy of edoxaban monotherapy after bioabsorbable polymer everolimus-eluting stent implantation in a human-like coronary atherosclerotic porcine model

**DOI:** 10.1016/j.athplu.2025.01.002

**Published:** 2025-01-31

**Authors:** Daisuke Kitano, Suguru Migita, Yuxin Li, Yutaka Koyama, Katsunori Fukumoto, Sayaka Shimodai-Yamada, Akira Onishi, Daiichiro Fuchimoto, Shunichi Suzuki, Yoshiyuki Nakamura, Atsushi Hirayama, Hiroyuki Hao, Yasuo Okumura

**Affiliations:** a Division of Cardiology, Department of Medicine, Nihon University School of Medicine, Tokyo, Japan; b Division of Advanced Cardiovascular Imaging, Department of Medicine, Nihon University School of Medicine, Tokyo, Japan; c Division of Human Pathology, Department of Pathology and Microbiology, Nihon University, Tokyo, Japan; d Division of Cell Regeneration and Transplantation, Department of Functional Morphology, Nihon University School of Medicine, Tokyo, Japan; e Department of Animal Science and Resources, College of Bioresource Sciences, Nihon University, Fujisawa, Japan; f Institute of Agrobiological Sciences, National Agriculture and Food Research Organization (NARO), Tsukuba, Japan; g Agricultural Technology Research Center, Swine and Poultry Research, Saitama, Japan; h Cardiovascular Division, Osaka Police Hospital, Osaka, Japan; i Internal Medicine, Osaka Fukujyuji Hospital, Osaka, Japan

**Keywords:** Atrial fibrillation, Edoxaban, Drug-eluting stent, Porcine model, Optical coherence tomography, Histology

## Abstract

**Background:**

The combination of antiplatelet and antithrombotic drugs increases the risk of bleeding in patients with atrial fibrillation after coronary drug-eluting stent (DES) implantation. However, the appropriateness of direct-acting oral anticoagulant (DOAC) monotherapy at the time of stent implantation remains uncertain. The objective of this study was to evaluate the safety and efficacy of DOAC monotherapy, specifically using factor Xa inhibitors such as edoxaban, in a low-density lipoprotein receptor knockout (LDL-R^−/−^) miniature pig model of human-like unstable coronary plaques compared to conventional dual-antiplatelet therapy (DAPT).

**Methods:**

We evaluated the safety and efficacy of edoxaban monotherapy in the LDL-R^−/−^ pig model with human-like unstable coronary plaques induced by a high-cholesterol, high-fat diet. Animals underwent DES implantation, followed by four weeks of treatment with either edoxaban monotherapy (3 mg/kg/day) or the DAPT regimen (aspirin 100 mg/day and clopidogrel 75 mg/day). Outcomes were assessed by optical coherence tomography (OCT), virtual histology intravascular ultrasound (iMap-IVUS), and histology. Key endpoints included in-stent thrombus formation, neointimal thickness, and coronary plaque composition.

**Results:**

Edoxaban monotherapy demonstrated a significantly thinner neointimal layer (120.0 [92.5–160.0] μm vs. 210.0 [180.0–240.0] μm, *p* < 0.001) and smaller neointimal area (1.06 [0.82–1.46] mm^2^ vs. 1.84 [1.61–2.24] mm^2^, *p* < 0.001) compared to DAPT. Neointimal coverage, fibrin deposition, and inflammatory cell infiltration were comparable between groups. No in-stent thrombi were observed in either group. iMap-IVUS findings indicated that edoxaban monotherapy significantly suppressed the increase in lipidic and necrotic plaque area while promoting fibrotic area expansion.

**Conclusions:**

Edoxaban monotherapy demonstrated superior efficacy in suppressing neointimal hyperplasia and stabilizing coronary plaques compared to DAPT with equivalent safety in preventing in-stent thrombus formation. These results provide important preclinical evidence supporting the potential of DOAC monotherapy as an antithrombotic strategy after DES implantation and warrant further investigation in clinical trials.

## Highlights

• Enhancing the relevance of translational research to simulate human coronary artery responses using a human-like coronary artery plaque porcine model.
• Equivalent safety of edoxaban monotherapy versus DAPT in preventing in-stent thrombus formation.
• Greater efficacy of edoxaban monotherapy compared to DAPT in reducing in-stent neointimal hyperplasia and stabilizing coronary artery plaque.

## 1 Introduction

Two decades have passed since the advent of drug-eluting stents for coronary artery disease. Advances in stent material, structure, and the drugs and polymers applied to the stents have significantly reduced the incidence of stent restenosis and thrombosis. Dual antiplatelet therapy (DAPT), combining aspirin and P2Y_12_ antagonists, is obligatory for deploying metallic stents in coronary arteries [1]. In recent years, the duration of antiplatelet therapy has been progressively shortened [[2], [3], [4]]. Conversely, the aging population has led to an increase in the number of patients with atrial fibrillation (AF). These individuals require oral anticoagulants (OACs) and are often prescribed both antiplatelet and OAC drugs when undergoing coronary artery stenting. However, triple therapy involving DAPT and warfarin carries a risk of bleeding [5].

Over the past decade, direct oral anticoagulants (DOACs) have emerged. Numerous clinical trials have demonstrated that dual therapy, combining antiplatelet drugs and DOACs, specifically factor Xa inhibitors or thrombin inhibitors, can reduce the risk of cardiovascular events in patients with AF undergoing percutaneous coronary intervention with metallic stents [[6], [7], [8]]. In addition, Yasuda et al. reported that DOAC monotherapy was sufficient after one year of stent implanation [9]. Consequently, a recent trend has emerged to recommend reduced and shorter antithrombotic therapy [2,3,10].

While it would be beneficial if DOAC monotherapy could be considered acceptable from the time of stent implantation in patients with coronary artery disease and AF, the safety and efficacy of this approach remain uncertain. This uncertainty stems from the formidable challenge of the validating the safety and efficacy of DOAC monotherapy in humans.

To address this gap, we have developed a low-density lipoprotein receptor knockout (LDL-R^−/−^) miniature pig model. These pigs, subjected to a high-cholesterol and high-fat (HCHF) diet for four months, exhibit human-like coronary plaques [11]. This animal model holds immense value because it allows experimental simulation of conditions and responses of coronary arteries that are difficult to verify in humans.

Therefore, the primary objective of this study is to determine the safety and efficacy of DOAC monotherapy, specifically edoxaban, from the time of DES implantation. We are using a porcine model with human-like coronary atherosclerotic plaques to evaluate in-stent thrombus response as a measure of safety and neointimal response as an indicator of efficacy.

## 2 Materials and methods

All animal care and experiments were performed in accordance with the National Institutes of Health Guidelines for the Care and Use of Laboratory Animals and the Basic Guidelines for Conduct of Animal Experiments published by the Ministry of Health, Labor and Welfare, Japan. The experimental protocol was approved by the Safety Committees for Gene Recombination Experiment of Nihon University (2020M8) and the Institutional Committee for the Use of Laboratory Animals of Nihon University School of Medicine (AP19MED030).

### 2.1 Animal preparation

**Fig. 1:**
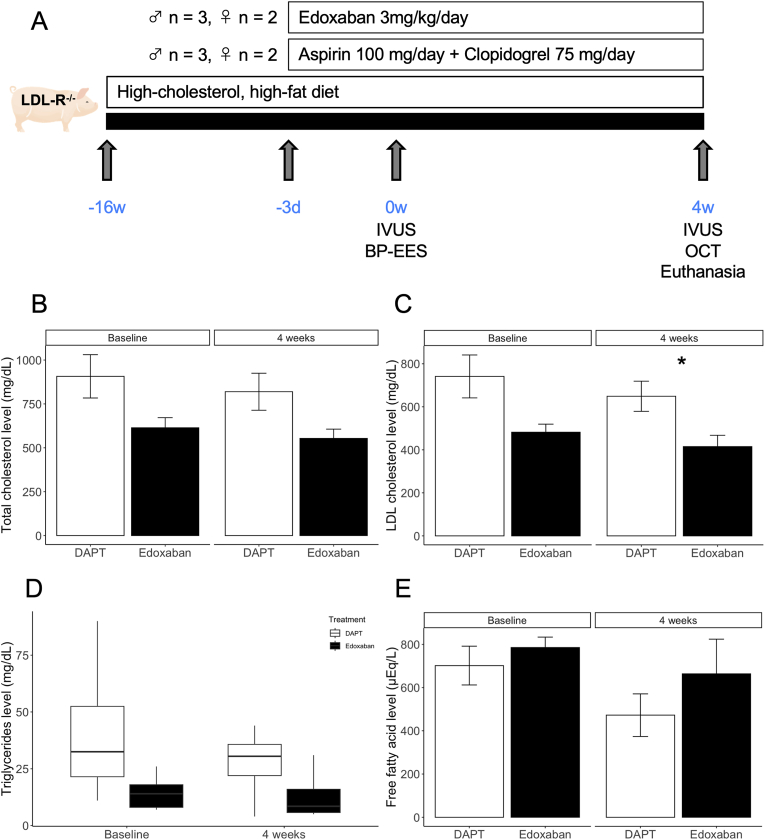
A, Outline of the low-density lipoprotein receptor knockout miniature pig experiment. B-E, Trends of lipid profiles between DAPT and edoxaban groups. Bar graphs show mean ± SE and the box plots show median and interquartile range. ∗, p < 0.05. LDL-R^−/−^, low-density lipoprotein receptor knockout; IVUS, intravascular ultrasound; BP-EES, bioabsorbable-polymer everolimus-eluting stent; OCT, optical coherence tomography; LDL, low-density lipoprotein.

The LDL-R^−/−^ miniature pigs with human-like advanced coronary plaque development used in this study were previously described in detail by Li et al. [11]. All animals were housed at the Nihon University Medical Research Support Center, and standard animal husbandry procedures were previously described [12] and followed. To accelerate the development of coronary artery plaques, 3-month-old male LDL-R^−/−^ miniature pigs were fed an HCHF diet (1 kg/day) throughout the experiment. The HCHF diet was prepared by adding 1.5 % cholesterol and 15 % powdered fat (Feed one Co., Ltd, Kanagawa, Japan) to standard pig chow. After 4 months of feeding the HCHF diet, the pigs were anesthetized with 3 % sevoflurane after sedation with midazolam (0.5 mg/kg, im). Using a general sterile technique, a 6F vascular sheath was inserted through the carotid artery, and heparin (100 IU/kg) was injected prior to catheterization. We performed coronary angiography and deployed bioabsorbable-polymer everolimus-eluting stents (SYNERGY stent, Boston Scientific, Marlborough, MA, USA) guided by intravascular ultrasound (IVUS) (OptiCross catheter and iLab system, Boston Scientific) (Fig. 1). A 3.0 × 20 mm or 3.5 × 20 mm stent was implanted in the right coronary artery and left anterior descending coronary artery. The stents were deployed at the site of the most intense atherosclerosis in each coronary artery. At the selected site, the balloon was inflated to achieve a stent-to-artery ratio of 1.1:1. Pigs received one of the following oral antithrombotic therapy regimens (for 4 weeks starting 3 days before stent implantation and until the end of the study): aspirin (100 mg/day) and clopidogrel (75 mg/day) (DAPT group, n = 5) or edoxaban (3 mg/kg/day) (edoxaban group, n = 5). A flowchart of the study protocol is shown in Fig. 1A. The dose of edoxaban was derived from our preliminary experiments (Supplementary Figure); the dose of 3 mg/kg/day in pigs is equivalent to the anticoagulant activity of 60 mg/day in humans [13]. These blood concentrations of edoxaban were measured by SNBL (Kagoshima, Japan). Edoxaban powder was provided as a gift by Daiichi Sankyo, Co., Ltd.

### 2.2 OCT analysis

At 4 weeks after stent implantation, OCT analysis was conducted following established procedures [14] using a frequency-domain OCT imaging system (C7 ILUMIEN OCT Intravascular Imaging Systems, Abbott Vascular, Santa Clara, CA, USA). In this study, a 2.7-F OCT imaging catheter (Dragonfly, Abbott Vascular) was advanced distal to the stented lesion, and an automated pullback was initiated to coincide with blood clearance facilitated by the injection of contrast media. The automatic pullback rate was 20 mm/s, with a frame rate of 100 frames/s.

OCT images were assessed using software (OPTIS, Abbott Vascular) as described previously [15]. Cross-sectional OCT images were analyzed at 1-mm intervals within the stented segment, 5 mm proximal and distal to the stent edges. Cross-sections featuring side branches or poor-quality OCT images were excluded from the analysis. Lumen and stent areas were delineated in each analyzed cross-section, and neointimal areas were calculated as [stent area–lumen area]. Neointimal thickness was measured exclusively at each stent strut, determined by automated measurements from the center of the luminal surface of each strut blooming to the lumen contour, as detailed previously [16].

### 2.3 iMap-IVUS analysis

Prior to stent implantation and at 4 weeks after stent implantation, IVUS was conducted. To assess the changes of native coronary artery wall with DOAC monotherapy, 10 mm proximal region to the stent implantation site, as measured by gray-scale IVUS, was analyzed in cross section at 1 mm each, using iMap-IVUS virtual histology software, specifically echoPlaque 4.0 (INDEC Medical Systems, Los Altos, CA, USA), as described previously [17]. In brief, vessel and lumen borders were delineated using automatic edge detection and manually corrected when necessary. Plaque components were then classified as fibrotic (light-green), lipidic (yellow), necrotic (pink) or calcified (light-blue). These components are presented as percentages (%) of the total plaque area.

### 2.4 Histological assessment

Following CAG and intracoronary imaging analysis, the pigs were immediately sacrificed. The coronary arteries were removed from the heart and whole stent segments were perfusion-fixed in 10 % buffered formalin for 48 h to wash away circulating blood components. then embedded in plastic with the strut. After polymerization of the plastic, stents were segmented at 3–5 mm intervals and histologic sections were cut at 5 μm with a tungsten carbide knife. The specimens were stained with hematoxylin-eosin or Masson's trichrome. Histological sections that had been damaged during the cutting or staining procedures were excluded.

Histological evaluation was performed as previously described [12]. The numbers of covered and total stent struts for the section were counted to calculate the ratio of struts covered by neointima. We considered stent struts to be covered if there was a microscopically detectable neointima over the struts. The neointimal thickness of the stent struts was defined as the distance from the margin of the struts to the luminal surface and was measured by image analysis software, ImageJ (ver. 1.52, National Institutes of Health, Bethesda, MD, USA) [18]. For all histological sections, obtained from porcine specimens, the area of fibrin deposition and the inflammation scores were evaluated. Area of fibrin deposition, defined as a reddish area around the stent struts on Masson's trichrome staining, was measured by ImageJ. The area of fibrin deposition was divided by the number of stent struts in each section, to facilitate comparison between the sections. Inflammation scores around the struts were semi-quantitatively graded by observation of 5 high power fields (HPF) per section. Scores were defined as follows: Grade 0, absent, few inflammatory cells except some infiltrating foreign body giant cells; Grade 1, mild, no more than 20 inflammatory cells; Grade 2, moderate, no more than 50 inflammatory cells; Grade 3, severe, more than 50 inflammatory cells per HPF. The maturity of neointima was evaluated in specimens harvested from the LDL-R^−/−^ miniature pigs. Mature neointima is considered to contain SMCs, which are eosin- and α-SMA-positive, while immature neointima is largely composed of extracellular matrix, containing proteoglycans which are alcian-blue-positive, with a few SMCs [19]. The assessment was performed for the neointima above the stent struts and that distant from the stent struts. We randomly selected one HPF of the neointima above the stent struts per section. The ratio of the eosin-stained area per neointimal area was calculated using ImageJ. Simultaneously, the ratios of the α-SMA-stained area and the alcian-blue stained area per neointimal area were calculated in the same way to evaluate the presence of SMCs and deposition of proteoglycans, respectively. Observation of Masson's trichrome clearly indicates the difference in extracellular matrix maturity, as compared to hematoxylin–eosin, at the area adjacent to the stent struts. The mature matrix is in the intima, while the immature matrix is in the neointima. Although identification of the border between the intima and neointima can be challenging in the area distant from the struts, we traced the border between the intima and neointima close to the struts to identify the border between them. Also, some struts remained uncovered. On the other hand, the degree of re-endothelialization of the neointima was evaluated. Lumen length and the region covered by endothelial cells, which are immunohistochemically CD31-positive, were measured for each section. The ratio of the re-endothelialized region was calculated by the following formula: length of re-endothelialized region/lumen length.

### 2.5 Statistical analysis

Data were presented as mean ± standard error of the mean (SEM) or median [interquartile range] for normally distributed and non-normally distributed variables, respectively. The Student's t-test or Mann-Whitney *U* test was used to compare differences between groups for continuous variables, and the chi-squared test or Fisher's exact test was used for categorical variables. All statistical analyses were performed using RStudio (version 2022.07.1, RStudio, Inc., Boston, MA, USA), an integrated development environment for R (version 4.2.1, The R Foundation for Statistical Computing, Vienna, Austria). A *p* value of less than 0.05 was considered statistically significant.

## 3 Results

### 3.1 Lipid profile of the study animal

Changes in blood lipid components over time are shown in Fig. 1B–E. LDL-R^−/−^ pigs fed the HCHF diet were severely hyperlipidemic. Baseline and 4-week total cholesterol, triglyceride, and free fatty acid levels were comparable across both groups. The edoxaban group had lower LDL cholesterol levels at 4 weeks.

### 3.2 OCT findings

**Fig. 2:**
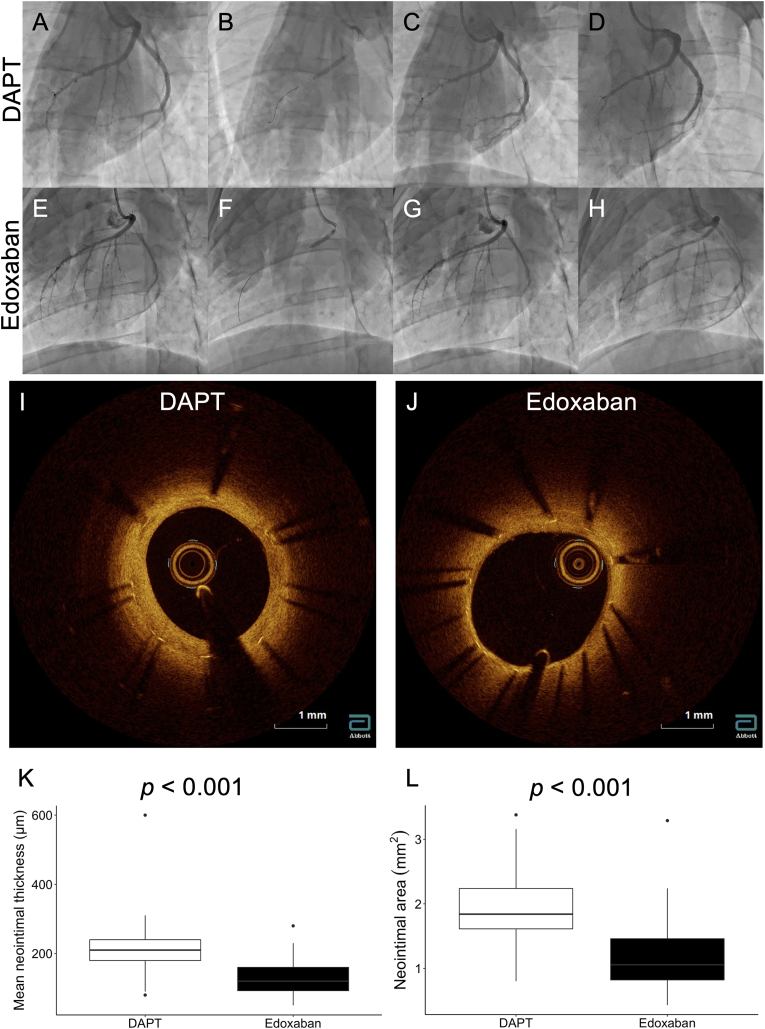
Representative cases and quantitative analysis of coronary angiography and optical coherence tomography findings. Coronary angiography images before stent implantation in the DAPT group (A) and the edoxaban group (E), during stent implantation in the DAPT group (B) and the edoxaban group (F), after stent implantation in the DAPT group (C) and the edoxaban group (G), and 4 weeks after stent implantation in the DATP group (D) and the edoxaban group (H). Cross-sectional image of a coronary artery 4 weeks after BP-EES implantation in the DAPT group (I) and the edoxaban group (J). K, Quantitative data of mean neointimal thickness between DAPT and edoxaban groups. L, Quantitative data of neointimal area between DAPT and edoxaban groups. DAPT, dual antiplatelet therapy.

As shown in Fig. 2, the stent struts were almost completely covered in both groups. The mean neointimal thickness in the edoxaban group was significantly thinner than that in the DAPT group (120.0 [92.5–160.0] μm vs. 210.0 [180.0–240.0] μm, *p* < 0.001). In addition, the neointimal area was significantly smaller in the edoxaban group than in the DAPT group (1.06 [0.82–1.46] mm^2^ vs. 1.84 [1.61–2.24] mm^2^, *p* < 0.001).

### 3.3 Histological findings

**Fig. 3:**
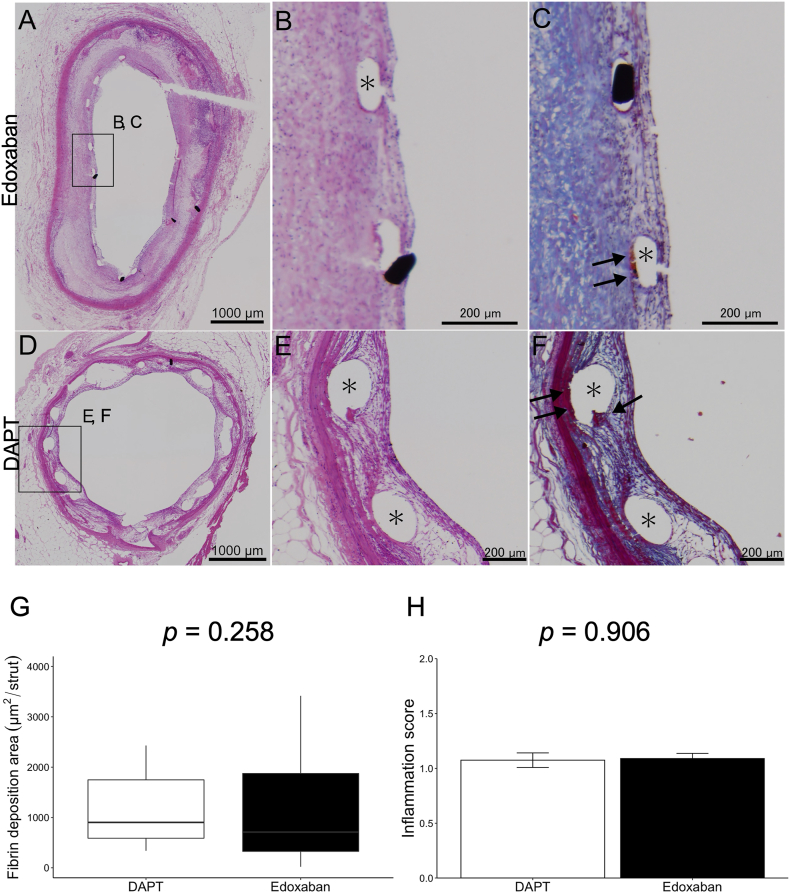
Representative cases and quantitative analysis of histological findings. A-C, Coronary artery walls treated with edoxaban for 4 weeks after BP-EES implantation. D-F, Coronary artery walls treated with DAPT for 4 weeks after BP-EES implantation. A, B and D, E shows tissues stained with hematoxylin and eosin, C and F show corresponding tissues stained with Masson's trichrome. G shows quantitative data of fibrin deposition area between DAPT and edoxaban groups. H shows quantitative data of inflammation score between DAPT and edoxaban groups. ∗ indicates stent strut. Arrows in C and F indicate fibrin deposition. DAPT, dual antiplatelet therapy consisting of aspirin and clopidogrel. BP-EES, bioabsorbable-polymer everolimus-eluting stent. BP-EES, bioabsorbable-polymer everolimus-eluting stent; DAPT, dual antiplatelet therapy.

The percentage of covered stent struts was comparable between the two groups (94.6 % in the edoxaban group vs. 97.7 % in the DAPT group, *p* = 0.364). As shown in Fig. 3, fibrin deposition around the stent struts was similar in both groups (709.7 [327.1–1874.9] μm^2^/strut in the edoxaban group vs. 1021.6 [678.3–2373.6] μm^2^/strut in the DAPT group, *p* = 0.258) (Fig. 3G). Inflammatory cell infiltration in the peri-strut region, expressed as a score, was not different between the two groups (1.09 ± 0.05 in the edoxaban group vs. 1.07 ± 0.07 in the DAPT group, *p* = 0.906) (Fig. 3H). No in-stent thrombi were observed in either group.

### 3.4 iMap-IVUS virtual histological findings

**Fig. 4:**
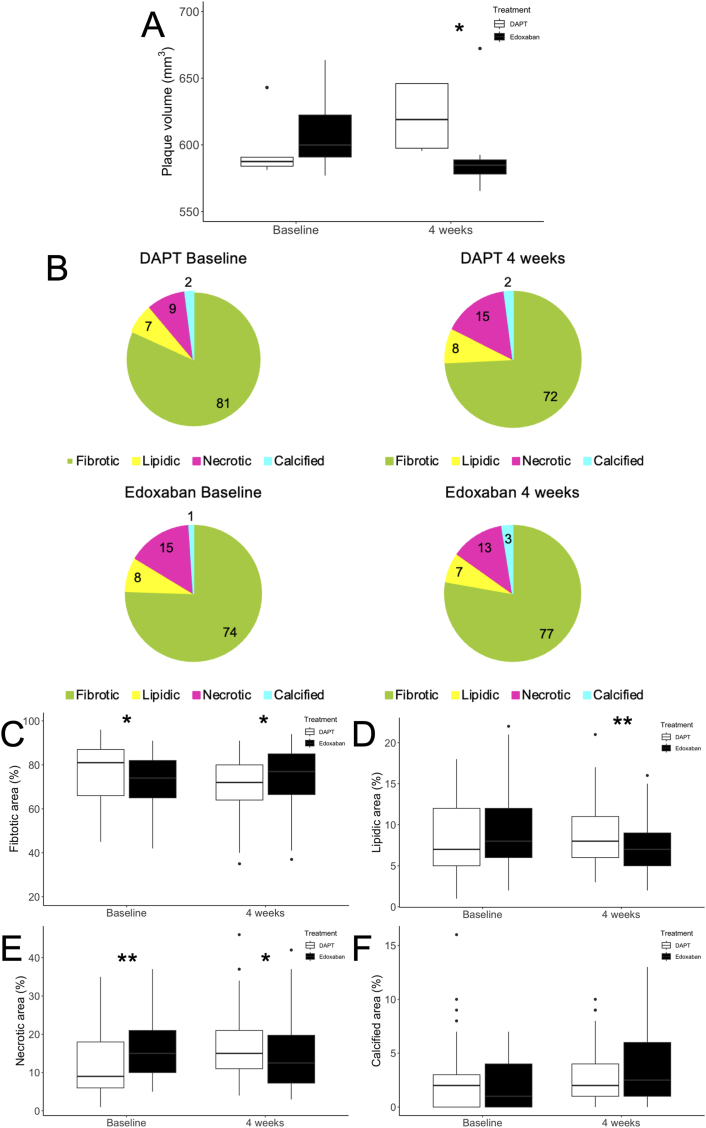
Effect of anti-thrombotic therapy with DAPT or edoxaban on atherosclerotic plaques. A, Change over time in native coronary plaque volume between 10 mm proximal to the stent as assessed by gray-scale IVUS. B, Tissue type as a percentage of area in the proximal region of the native coronary artery wall using iMap-IVUS virtual histologic analysis and changes before and after anti-thrombotic therapy. Comparison of each component of the atherosclerotic coronary artery wall is shown as box plot: C) fibrotic area; D) lipidic area; E) necrotic area; and F) calcified area. Box plots show median and interquartile range. ∗, p < 0.05; ∗∗, p < 0.01. DAPT, dual antiplatelet therapy; IVUS, intravascular ultrasound.

After 4 weeks of administration of either DAPT or edoxaban, the plaque volume in the native coronary artery wall group was significantly reduced in the edoxaban group (584.8 [578.1–588.8] mm^3^ vs. 619.0 [597.5–646.0] mm^3^, *p* = 0.048) (Fig. 4A). Fig. 4B shows the atherosclerotic content of the native coronary artery wall. After 4 weeks of edoxaban treatment, the increase in lipidic and necrotic plaque area was significantly suppressed (lipidic area, 7.0 % vs. 8.0 %, *p* < 0.001; necrotic area, 12.5 % vs. 15.0 %, *p* = 0.014). Conversely, the fibrotic area increased significantly after 4 weeks of edoxaban treatment (77.0 % vs. 72.0 %, *p* = 0.022) (Fig. 4C–F).

## 4 Discussions

This animal study demonstrated the in-stent responses to edoxaban monotherapy, a factor Xa inhibitor, after DES implantation, using both intracoronary imaging analysis and histologic examination. The results of the study provide evidence of the safety of edoxaban monotherapy from the time of DES implantation. While previous studies have suggested that edoxaban monotherapy may reduce the incidence of stent thrombosis in small animal models [20], the present study demonstrates that edoxaban monotherapy is as safe as DAPT in preventing stent thrombosis in a large animal model. The advantages of conducting large animal experiments include the large size of the coronary arteries and the similarity of the porcine cardiovascular system to that of humans, making this model particularly suitable for studying coronary interventions. These factors underscore the translational relevance of our findings and highlight the novelty of extending research from small animal models to a large animal context. In addition, edoxaban monotherapy was shown to be effective in inhibiting neointimal progression after DES implantation.

Large clinical trials have demonstrated the efficacy and safety of DOAC monotherapy after PCI. In a recent retrospective study, Hwang et al. evaluated the efficacy of DOAC monotherapy switching one year after percutaneous coronary intervention (PCI) [21]. The MACE incidence of DOAC monotherapy did not differ from that of combination therapy (DOAC and an antiplatelet agent). Fukamachi et al. in the PRAEDO AF trial showed that edoxaban monotherapy has acceptable clinical safety in patients with AF and stable CAD compared with edoxaban plus clopidogrel treatment [22]. In addition, the AFIRE, OAC-ALONE, and EPIC-CAD trials, which randomized patients with AF and CAD to DOACs alone or in combination with an antiplatelet agent, showed a lower risk of composite events [9,23,24]. In this animal study, no apparent in-stent thrombus was observed after stent implantation, even with edoxaban monotherapy. Mechanical injury to the vessel wall activates the coagulation system and generates factor Xa and thrombin [25,26]. In addition, exposure of blood flow to the stent struts causes turbulence and activates the coagulation system [27]. Therefore, the inhibition of factor Xa after stent placement may be effective in preventing stent thrombus formation. Previous preclinical studies have shown that factor Xa inhibitors impair platelet activation and inhibit stent thrombus formation [20,28,29]. These findings suggest that factor Xa inhibitor monotherapy without antiplatelet agents from the time of stent implantaion may be appropriate from a safety perspective.

Regarding neointimal hyperplasia after stent implantation, as we have previously reported, the neointima is relatively thin after third-generation DES implantation in the porcine model [12]. Interestingly, the neointima was even thinner in the edoxaban monotherapy group. As mentioned above, vascular injury activates coagulation cascades, and these coagulation factors play important roles in cellular responses such as inflammation and proliferation through the PARs pathway [30,31]. In addition, factor Xa and PAR2 have been documented to play a pivotal role in the proliferation of vascular smooth muscle cells [[32], [33], [34], [35], [36]] and neointimal formation [[37], [38], [39]]. Previous preclinical studies have demonstrated that edoxaban treatment prevents maladaptive vascular remodeling [[40], [41], [42]]. Factor Xa inhibitors are known to play an important role in suppressing the progression of atherosclerosis via PAR2 [43,44]. In the present study, the neointimal coverage after stenting was comparable to that with DAPT. In addition, native coronary plaque lesions exhibited enhanced stabilization in the edoxaban monotherapy group. Considering the above, we believe that the efficacy of edoxaban monotherapy after stent implantation is superior to that of DAPT. Although our results and previous evidence support DOAC monotherapy afterPCI, Marazzato et al. emphasize that reducing the intensity of antithrombotic therapy should be considered with caution in patients at high risk of ischemic events, such as those with acute coronary syndrome, residual untreated coronary lesions, and complex PCI lesions [45].

Our study has several limitations. Because only a single dose of edoxaban was examined in this study, the dose-response relationship for edoxaban in the porcine cardiovascular system remains unclear. Biological responses differ between pigs and humans, particularly the response in pigs is faster than that in humans. Regarding the small sample size, we acknowledge the concern about statistical power. This number was chosen based on similar preclinical studies to balance ethical considerations with statistical requirements. The results were statistically significant despite the small sample size of this translational study. Because immunohistochemical and molecular cell biological analyses were not performed in this study, the molecular biological mechanisms by which edoxaban inhibits thrombus formation and suppresses neointimal growth after stent implantation remain to be elucidated.

## 5 Conclusions

Our results showed that the edoxaban monotherapy was non-inferior to DAPT after BP-EES implantation in terms of safety, such as in-stent thrombus formation, and suggested that edoxaban monotherapy was superior to DAPT in terms of re-endothelialization. In addition, edoxaban monotherapy appears to contribute to coronary plaque stabilization. These results provide as important evidence for an anti-thrombotic regimen after DES implantation.

## Funding sources

This work was partly supported by a research foundation from 10.13039/501100002336Daiichi Sankyo Co., Ltd. The funder had no role in finalizing the study design, data collection and analysis, and decision to publish. This work was supported by 10.13039/501100001691JSPS KAKENHI Grant Numbers JP19K16621 (DK), JP17K09595 (YL), JP16K08676 (HH), and the 10.13039/100007683Nihon University School of Medicine, Alumni Association 60th anniversary Fund Scholarship (2017) (DK).

## Declaration of competing interest

The authors declare the following financial interests/personal relationships which may be considered as potential competing interests: Dr. Hirayama received research funding from 10.13039/100004326Bayer Healthcare, 10.13039/501100022274Daiichi Sankyo, 10.13039/501100004948Astellas Pharma, 10.13039/501100003769Eisai, 10.13039/501100022074Sumitomo Dainippon Pharma, 10.13039/100030732MSD, 10.13039/100015377Nihon Medi-Physics, 10.13039/100002491Bristol-Meyers Squibb, Boehringer Ingelheim, and 10.13039/100004319Pfizer, and accepted remuneration from 10.13039/100004326Bayer, 10.13039/501100022274Daiichi Sankyo, 10.13039/501100003769Eisai, 10.13039/100002491Bristol-Meyers Squibb, 10.13039/501100004948Astellas, 10.13039/100004339Sanofi, and 10.13039/100008373Takeda. Dr. Okumura has received lecture fees from 10.13039/100015731Bayer Yakuhin and 10.13039/501100002336Daiichi Sankyo; research funding from 10.13039/100015731Bayer Yakuhin and 10.13039/100002491Bristol-Myers Squibb; and scholarship grants from 10.13039/100015731Bayer Yakuhin, 10.13039/501100022274Daiichi Sankyo, and 10.13039/100004331Johnson & Johnson.
