# Supplementary figures and images for: Safety and efficacy of edoxaban monotherapy after bioabsorbable polymer everolimus-eluting stent implantation in a human-like coronary atherosclerotic porcine model
Source: Atheroscler Plus. 2025 Jan 31;59:59–67. doi: 10.1016/j.athplu.2025.01.002 (PMC11848492; doi:10.1016/j.athplu.2025.01.002)

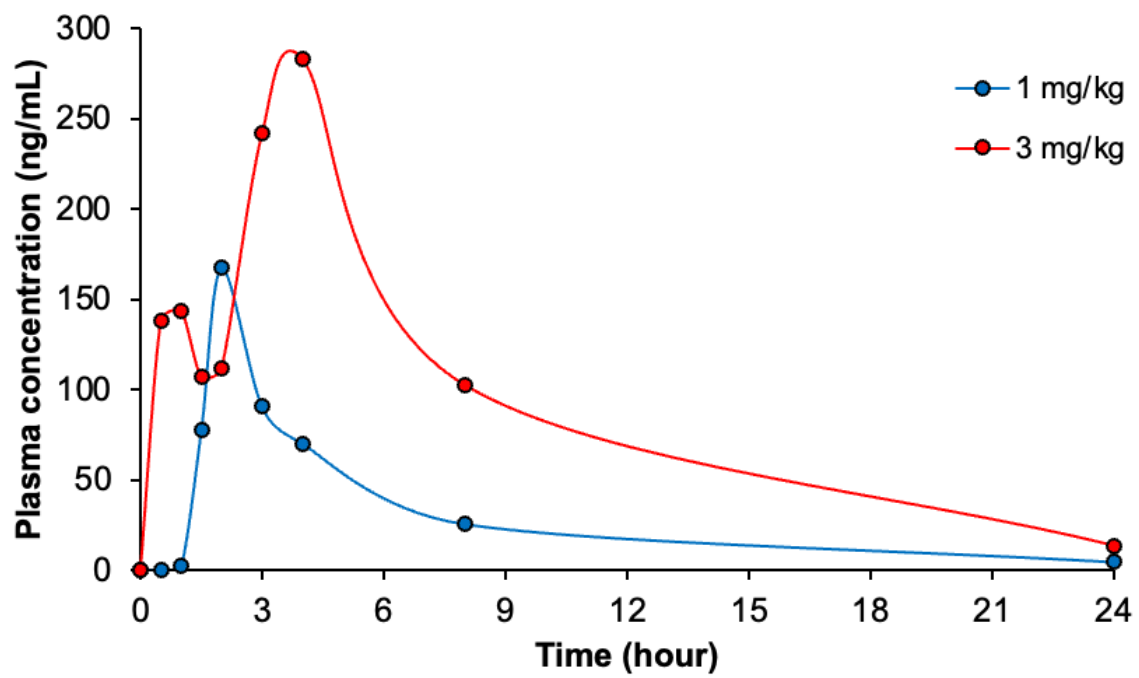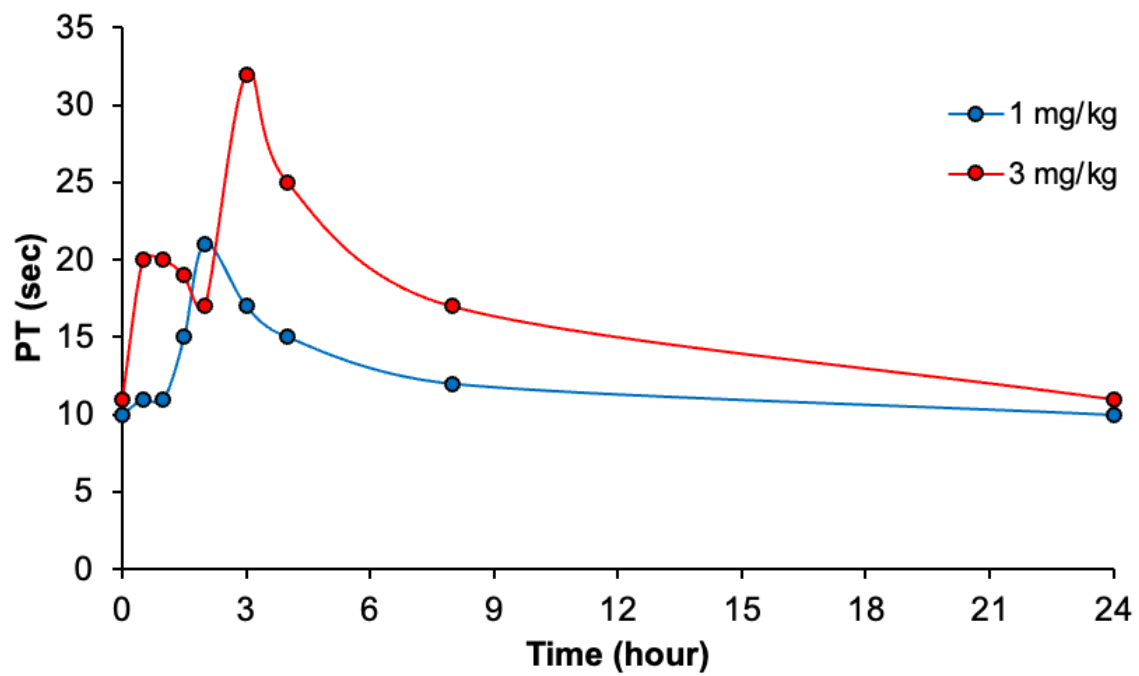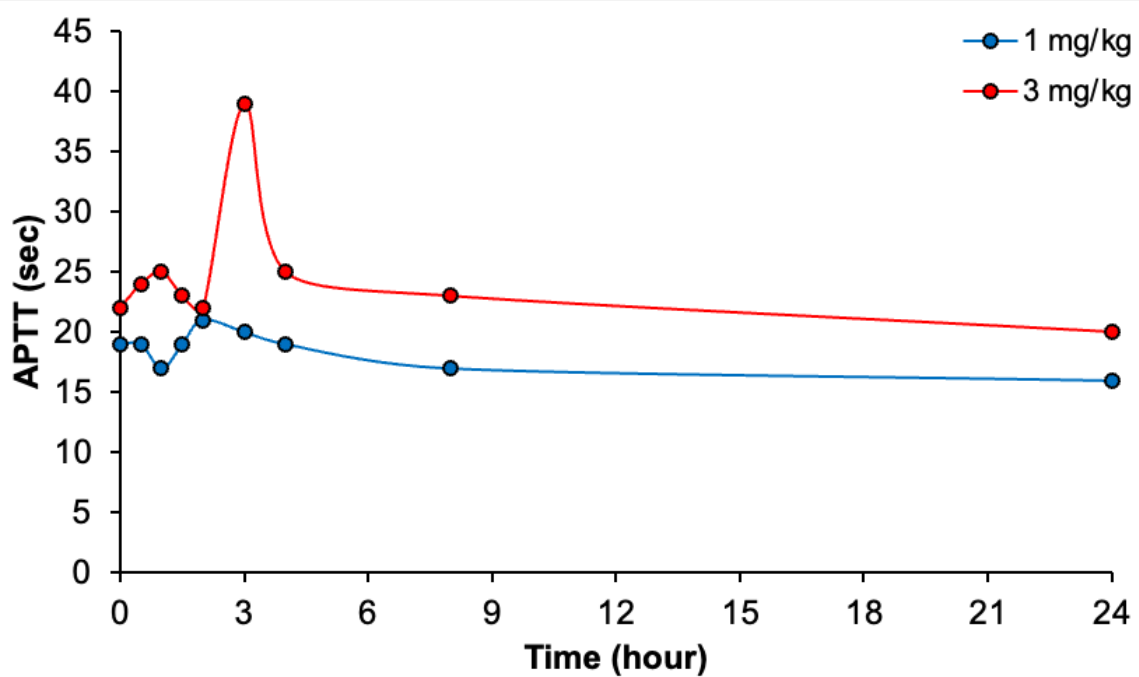

Supplement: Multimedia component 1 [file mmc1.pdf]
